# Neural Basis of the Time Window for Subjective Motor-Auditory Integration

**DOI:** 10.3389/fnhum.2015.00688

**Published:** 2016-01-07

**Authors:** Koichi Toida, Kanako Ueno, Sotaro Shimada

**Affiliations:** ^1^Department of Architecture, School of Science and Technology, Meiji UniversityKawasaki, Japan; ^2^Japan Science and Technology Agency, Core Research for Evolutionary Science and Technology (CREST)Saitama, Japan; ^3^Department of Electronics and Bioinformatics, School of Science and Technology, Meiji UniversityKawasaki, Japan

**Keywords:** delayed auditory feedback, event-related potential (ERP), enhanced-P2, N300, sense of agency

## Abstract

Temporal contiguity between an action and corresponding auditory feedback is crucial to the perception of self-generated sound. However, the neural mechanisms underlying motor–auditory temporal integration are unclear. Here, we conducted four experiments with an oddball paradigm to examine the specific event-related potentials (ERPs) elicited by delayed auditory feedback for a self-generated action. The first experiment confirmed that a pitch-deviant auditory stimulus elicits mismatch negativity (MMN) and P300, both when it is generated passively and by the participant’s action. In our second and third experiments, we investigated the ERP components elicited by delayed auditory feedback for a self-generated action. We found that delayed auditory feedback elicited an enhancement of P2 (enhanced-P2) and a N300 component, which were apparently different from the MMN and P300 components observed in the first experiment. We further investigated the sensitivity of the enhanced-P2 and N300 to delay length in our fourth experiment. Strikingly, the amplitude of the N300 increased as a function of the delay length. Additionally, the N300 amplitude was significantly correlated with the conscious detection of the delay (the 50% detection point was around 200 ms), and hence reduction in the feeling of authorship of the sound (the sense of agency). In contrast, the enhanced-P2 was most prominent in short-delay (≤200 ms) conditions and diminished in long-delay conditions. Our results suggest that different neural mechanisms are employed for the processing of temporally deviant and pitch-deviant auditory feedback. Additionally, the temporal window for subjective motor–auditory integration is likely about 200 ms, as indicated by these auditory ERP components.

## Introduction

Predicting the timing and occurrence of an auditory stimulus is a central feature of auditory processing (Haggard et al., 2002; Sugita and Suzuki, 2003; Fujisaki et al., 2004; Vroomen et al., 2004; Fujisaki and Nishida, 2010; Yamamoto et al., 2012). This is particularly important when a sound is made by an individual’s own actions, such as those involved in playing a musical instrument, operating machinery, dancing, vocalization, and everyday physical movement. Simultaneous judgment of a sound and a corresponding action is thought to be based on predictive processing of upcoming auditory stimuli using the internal forward model (Wolpert et al., 1995; Miall and Wolpert, 1996; Wolpert and Ghahramani, 2000; Haggard et al., 2002). Auditory feedback can be delayed, either by a physical constraint of a musical instrument or mechanical tool, or simply by physical distance. Thus, the human brain is endowed with a sensorimotor integration mechanism that can absorb subtle differences in timing between a self-generated action and the resulting auditory feedback. Indeed, our recent study showed that auditory feedback that is delayed by 200–300 ms can be perceived as simultaneous in an experimental setting where the auditory feedback is elicited by an individual’s simple action (Toida et al., 2014). However, the neural mechanisms that underlie the temporal integration of a self-action and the corresponding auditory feedback are not fully understood.

The mismatch negativity (MMN) is a frontocentrally distributed negativity obtained by subtracting the event-related potential (ERP) waveform elicited during passive listening of a standard (frequent) auditory stimulus from that of a deviant (rare) stimulus. The MMN typically peaks 150–200 ms after stimulus onset (Sams et al., 1984; Näätänen et al., 1997, 2001, 2010). The MMN is evoked by noticeable differences in not only the physical features of an auditory stimulus, such as the pitch, stimulus length, and power, but also in the more abstract features, such as the sequential pattern of auditory stimuli (Bendixen et al., 2012; Paavilainen, 2013). This indicates that auditory processing involves the prediction of upcoming auditory inputs, such that a violation to this prediction elicits a MMN (Joos et al., 2014). Although many studies have examined the conditions that induce the MMN, to the best of our knowledge, no previous studies have focused on whether a temporally deviant (delayed) auditory self-initiated stimulus also elicits a MMN.

Here we report that violating the timing expectation of auditory stimuli elicits specific ERP components, the enhanced-P2 and the N300, that can be differentiated from the MMN. Notably, these components showed modulation as a function of the delay length. In this study we conducted four experiments with an oddball experimental paradigm while measuring by electroencephalogram (EEG). In the first experiment, we sought to confirm that a pitch-deviant auditory stimulus elicits a MMN regardless of whether the auditory stimulus was generated by the participant’s action (Experiment 1). In the second experiment, we examined the ERP components elicited by delayed auditory feedback produced by a self-generated action (Experiment 2). In the third experiment, we used a mixed (pitch-delay) experimental design to further differentiate the characteristics of the ERP components elicited by delayed auditory feedback from those of the MMN (Experiment 3). In the final experiment we examined the sensitivity of the ERP components to the delay length (Experiment 4). Our findings indicate that delayed auditory feedback substantially modulates the neural processing that occurs within a few 100 ms from the onset of auditory feedback.

## Materials and Methods

### Participants

Sixty-four healthy students participated in the experiment (eight females and eight males for each of the first, second, third, and fourth experiments; mean age 20.9 ± 1.4, range 18–25). A one-way analysis of variance (ANOVA) demonstrated that the ages of the participants were not significantly different among experiments (*F* < 1, not significant). Two additional individuals participated in Experiment 3, but they were excluded from the analysis owing to difficulties with the EEG measurement (one) and poor behavioral performance (one; target detection rate was 53.3%; Smirnov–Grubbs outlier test, *p* < 0.05). The participants received monetary compensation. All participants were right-handed, had normal hearing, and had no history of neuropsychiatric disorders or neurological surgery. The participants were unaware of the purpose of the experiment. The experiments were approved by the ethics committee of the School of Science and Technology, Meiji University, and conducted according to the principles and guidelines of the Declaration of Helsinki. Written informed consent was obtained from all participants.

### EEG Recordings

Electroencephalogram were recorded from Ag/AgCl active electrodes, with a sampling rate of 512 Hz, and band-pass filtered at 0.5–30 Hz. Electrodes were placed on four midline sites, Fpz, Fz, Cz, and Pz, according to the international 10–20 system, and embedded in an elasticized cap montage. The reference electrode was placed at the left earlobe. Electrooculograms (EOG) were also recorded via an electrode attached 20 mm above the left eye. Electrode impedances were kept under 10 kΩ. The EEG and EOG were recorded using a biosignal amplification unit (g.USBamp, g.tec medical engineering GmbH, Schiedlberg, Austria).

### Experiment 1: ERPs Elicited by Pitch-Deviant Auditory Feedback

**FIGURE 1:**
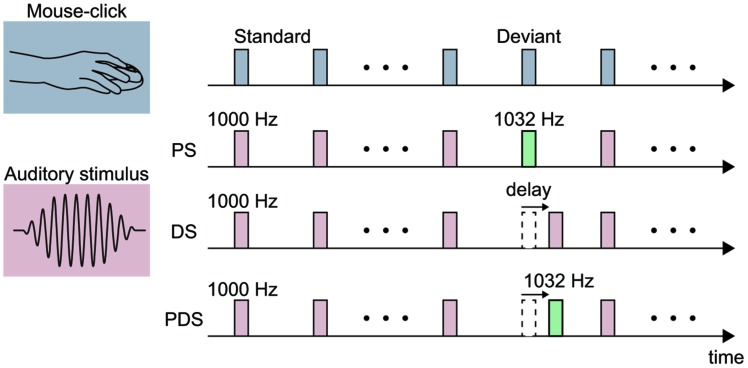
**Schematic illustration of the experimental stimuli.** Each stimulus was either elicited by a mouse-click performed by the participant (action condition; top) or presented passively with a fixed (1 s) interval (non-action condition in Experiments 1 and 2). A pitch-deviant stimulus (PS) was used in Experiments 1 and 3. The PS was a 1032-Hz pure tone (presentation rate 20%) and the standard stimulus was a 1000-Hz pure tone (80%). Delayed stimuli (DS) were used in Experiments 2–4. The delay length was fixed at 150 ms in Experiments 2 and 3, and varied from 100 to 400 ms in Experiment 4. A pitch-deviant delayed stimulus (PDS) was used in Experiment 3. Specifically, this was a 1032-Hz pure tone presented with a 150 ms delay with respect to the mouse-click.

In Experiment 1, we examined ERPs elicited by a pitch-deviant auditory stimulus. We employed the standard oddball experimental paradigm with a 1000-Hz pure tone as the standard stimulus and a 1032-Hz pure tone as a deviant stimulus (**Figure 1**). Both stimuli had durations of 30 ms and faded in/out for 10 ms. The stimuli were presented at a comfortable listening level. The standard stimulus was presented 360 times (80%) and the deviant was presented 90 times (20%) in each session. The deviant stimulus was not presented consecutively.

The auditory stimulus was presented either passively (non-action condition), as in previous MMN studies (non-action condition; Joos et al., 2014), or in association with a mouse-click movement made by the participant (action condition). The intertrial interval (ITI) was set at 1000 ms in the non-action condition. In the action condition, the participant made a series of mouse-clicks separated by approximately 1000-ms. This action was practiced prior to the experiment until an appropriate performance was achieved. The average ITI in the action condition was 1081.7 ± 99.9 ms. Participants were instructed to silently count the number of pitch-deviant auditory stimuli they could detect, and to report this value at the end of the session. The average number of reported deviant stimuli was similar among the conditions [84.2 ± 10.1 in the non-action condition and 81.4 ± 13.8 in the action condition; the deviant stimulus was presented 90 times; *t*_(15)_ = -1.31, *p* = 0.21, effect size *r* = 0.32]. Each condition (action or non-action) was conducted in one session, and the order of sessions was counterbalanced across participants.

We ran the experiments on a built-to-order PC using E-Prime 2.0 (Psychology Software Tools Inc., Sharpsburg, PA, USA). In a soundproof room, the participants sat at a desk at a distance of 1 m from a cross-shaped fixation point on the wall, wearing in-ear monitor earphones (ER-4B, Etymotic Research, Elk Grove Village, IL, USA). A computer mouse specially designed for high-speed gaming (Gaming Mouse G500, Logicool, Tokyo, Japan) was located on the desk. The direct mouse-click sound and the sound delivered through the earphones were simultaneously recorded, and the timing difference, that is, the intrinsic delay (the minimum delay from mouse-click to sound production) of our experimental setup, was calculated using a sound waveform viewer (Adobe Audition CS6, Adobe Systems Inc., Mountain View, CA, USA). We repeated this procedure 20 times and found that the intrinsic delay was 53.3 ± 2.5 ms.

### Experiment 2: ERPs Elicited by Delayed Auditory Feedback

In Experiment 2, we investigated the ERP components elicited by delayed auditory feedback of the self-movement. To this end, we employed an oddball paradigm with a temporally deviant stimulus (**Figure 1**). The delay of the deviant stimulus was set to 150 ms in this experiment. In the non-action condition, the standard and deviant stimuli were delivered with ITIs of 1000 and 1150 ms, respectively. The average ITIs for the standard and deviant stimuli in the action condition were 1081.2 ± 147.8 and 1167.9 ± 157.6 ms, respectively.

In the action condition, the deviant stimulus was delivered with a 150 ms delay after the participant performed a mouse-click, while the standard stimulus was not delayed. The auditory feedback about the mouse-click in the action condition included the intrinsic delay of 53 ms, such that the effective delays for the standard and deviant stimuli were 53 and 203 ms, respectively. Both the standard and deviant stimuli were 1000-Hz pure tones. Participants were instructed to silently count the number of trials in which they could detect a delayed auditory stimulus, presented either passively (non-action condition) or in association with their mouse-click movement (action condition). The average number of detected deviant stimuli was similar between conditions [73.6 ± 9.3 in the non-action condition and 78.5 ± 11.8 in the action condition, where the deviant stimulus was presented 90 times; *t*_(15)_ = -2.00, *p* = 0.06, effect size *r* = 0.46]. Each condition was conducted in one session, and the order of sessions was counterbalanced across participants. The other experimental settings were identical to those in Experiment 1.

### Experiment 3: Functional Dissociation of ERPs Elicited by Pitch-Deviant and Delayed Auditory Feedback

In Experiment 3, we sought to further elucidate the characteristics of ERPs elicited by pitch-deviant and delayed auditory feedback. To this end, we conducted a mixed (pitch-delay) design experiment with three types of deviant stimuli (**Figure 1**). The standard stimulus was a 1000-Hz pure tone that was not delayed (except for the intrinsic delay of 53 ms). There were three types of deviant stimuli: (1) 1032-Hz pitch, non-delayed (pitch-deviant stimulus, PS); (2) 1000-Hz pitch, 150-ms delayed (delayed stimulus, DS); and (3) 1032-Hz pitch, 150-ms delayed (pitch-deviant delayed stimulus; PDS). The standard stimulus was presented 1080 times (80%) whereas the three types of deviant stimuli were presented 90 times each (for a total of 270 times between the three deviant stimuli, 20%) in each session. The presentation order of the three types of deviant stimuli was pseudo-random. We hypothesized that the PDS would elicit pitch-deviant ERPs if the processing of pitch-deviant feedback dominates that of delayed feedback. Correspondingly, if the processing of delayed feedback dominates that of pitch-deviant feedback, then we would expect the PDS to elicit delayed ERPs. Alternatively, if pitch-deviant feedback and delayed auditory feedback are processed independently by different neural mechanisms, then we would expect to observe a mixture of delayed and pitch-deviant ERPs.

In this experiment, the auditory stimuli were elicited by the mouse-click action performed by the participants (action condition only), as our main aim was to elucidate the neural processes underlying delayed auditory feedback of self-action and the ERPs tended to be more enhanced in the action condition in Experiments 1 and 2 (see Results). At the beginning of the session, the participants were instructed to count the number of trials where they detected either pitch-deviant (attend-to-pitch session) or delay-deviant (attend-to-delay session) auditory feedback. Note that the target stimuli were PS and PDS but not DS in the attend-to-pitch session, while they were DS and PDS but not PS in the attend-to-delay session. This manipulation enabled us to examine the effects of attention on the elicited ERP components. Before the experiment, participants practiced discriminating the pitch-deviant and delay-deviant stimulus from the standard stimulus until adequate performance was observed. The other experimental settings were identical to Experiment 1.

### Experiment 4: Sensitivity to Delay Length

In Experiment 4, we further investigated the sensitivity of ERP components for delayed auditory feedback to the length of the delay. The standard stimulus was a 1000-Hz pure tone that was not delayed. There was a control condition (standard stimulus only) and four delay conditions in which the deviant stimulus (1000-Hz pure tone) was delayed by 100, 200, 300, or 400 ms. The control condition was introduced to obtain a baseline score regarding the sense of agency (see below). The standard stimulus was presented 120 times (80%) and the deviant was presented 30 times (20%) in one experimental block. Each condition was conducted in a separate block. One session comprised five blocks (five conditions) and three sessions were conducted for each participant (the total number of deviants was 90 for each condition). The order of conditions was pseudo-randomized across sessions and participants.

In this experiment, only the action condition was performed. Participants were required to count the number of trials in which they could detect the delayed auditory feedback. To examine the delay detection threshold (DDT, detection of feedback delay at 50% probability), logistic curves were fitted to the participant responses on the basis of the following formula (Toida et al., 2014):

$$P\left(t\right)=\frac{1}{1+\exp\left[-a\left(t-t_{DDT}\right)\right]}$$

where *t* is the auditory feedback delay length, *P*(t) is the probability of making a delay detection, *a* indicates the steepness of the fitted curve, and *t*_DDT_ indicates the observer’s point of subjective simultaneity (DDT). In our experiment, *t* served as an independent variable and *P*(t) was the observed value. Fitting was performed using a non-linear least squares method (a trust-region algorithm), provided by the Curve Fitting toolbox in MATLAB R2013b (MathWorks, Natick, MA, USA), to estimate *a* and *t*_DDT_.

We also assessed the sense of agency (Gallagher, 2000, 2005; Sato and Yasuda, 2005; Kalckert and Ehrsson, 2012), which is the feeling of authorship of an action, for each condition via four questions that were presented after each block. These were: “Did you feel like the sound was made by yourself?” (Q1), “Was the sound elicited in the way that you thought?” (Q2), “Did you feel like your hand was controlled by the sound?” (Q3, dummy question), and “Did you feel like your mind was controlled by the sound?” (Q4, dummy question), as per the previous study (Kalckert and Ehrsson, 2012). The participants used a seven-point Likert scale ranging from -3 (strongly disagree) to +3 (strongly agree) to respond. Other experimental settings were identical to those in Experiment 1.

### ERP Methods

We computed ERP waveforms for each participant by averaging the epoch from 300 ms before the auditory stimulus onset to 700 ms after the onset, including a 200 ms pre-stimulus baseline. Additionally, we computed ERP waveforms by averaging the epoch from 300 ms before the mouse-click onset to 1,000 ms after the onset, including a 200 ms pre-stimulus (before mouse-click) baseline in Experiments 2–4. The averaged ERP waveforms were obtained separately for each stimulus and electrode. The trials in which the EEG exceeded ±80 μV were rejected from the ERP averaging. We used independent component analysis (ICA) to eliminate ocular artifacts in the EEG data. The ICA component with the most significant correlation with the EOG data was removed. The remaining data were back-projected to create EEG signals. We then calculated grand-averaged ERP waveforms and differential (target – standard) ERP waveforms for each condition and each participant.

Based on preliminary ERP data, we focused on the characteristic ERP components elicited by each stimulus, namely the MMN and P300 for the pitch-deviant stimuli and the enhanced-P2 (see Results) and N300 components for the delayed stimuli. Incidentally, the ERP component preceding the MMN and the enhanced-P2 elicitation, namely auditory-N1 (negative peak around 100 ms; Hillyard et al., 1973; Hyde, 1997), was not significantly different among conditions in all experiments (*P* > 0.1), and thus we do not further report N1 results here. The MMN and enhanced-P2 components were designated as the largest negative and positive deflection peak between 150 and 250 ms after the auditory stimulus onset in the differential (target – standard) ERP waveforms, respectively (Ritter et al., 1982; Sams et al., 1985; Näätänen et al., 2007; Legrain et al., 2009). The individual amplitudes of MMN and enhanced-P2 were calculated as a mean voltage at the 40 ms period centered at the peak latencies in the grand-averaged differential ERP waveforms (Näätänen et al., 2004). The individual MMN and enhanced-P2 peak latencies were also measured from the most negative and positive deflection peak occurring at 150–250 ms from the onset. The P300 and N300 components were set as the largest positive and negative deflection peak between 250 and 450 ms after the auditory stimulus onset in the target ERP waveform, respectively. The time windows of the P300 and N300 components were decided by referring to previous studies (Duncan-Johnson and Donchin, 1982; Picton, 1992; Laszlo and Federmeier, 2012). In Experiment 3, we calculated both the MMN and enhanced-P2 components, and the component with the larger absolute amplitude in the differential waveform was submitted to the analyses. This procedure was also applied to the P/N300 components. For the differential (target – standard) ERP waveforms with the mouse-click onset, the omission MMN was designated as the largest negative deflection peak between 50 and 300 ms after the action onset (May and Tiitinen, 2010).

To assess the elicitation of ERP components in Experiments 1 and 2, we submitted the latency and amplitude of each component at each electrode to a one-sample *t*-test. Similarly, to assess the conditional differences (action vs. non-action), we submitted the latency and amplitude of each component at each electrode to a two-tailed paired *t*-test. In Experiment 3, we submitted the latency and amplitude of each ERP component to a two-way repeated measures ANOVA using the sessions (attend-to-pitch and attend-to-delay) and conditions (PS, DS, and PDS) as factors. When appropriate, we applied the Greenhouse–Geisser correction, 𝜀, for violation of sphericity. Any observed significant effect of a stimulus was followed by a *post hoc* comparison using Tukey’s honestly significant difference (HSD) test. In Experiment 4, we submitted the latency and amplitude of each ERP component to a one-way repeated measures ANOVA with the conditions (control, 100-ms-, 200-ms-, 300-ms-, and 400-ms-delayed) as factors. A significant effect of condition was tested via a *post hoc* comparison using Tukey’s HSD test. Additionally, we conducted linear regression analyses using the delay length as the explanatory variable and the Curve Fitting toolbox in MATLAB R2013b.

The normality of the data for all *t*-tests and correlation tests was confirmed by the Shapiro–Wilk test (*p* > 0.05). If the normality of the data did not hold true, we applied Welch’s *t*-test or Spearman’s rank correlation test, respectively. We used a false discovery rate control for the multiple comparisons adjustment in the peak analyses. The significance level was set at *p* < 0.05 for all statistical tests. We calculated the effect size *r* for *t*-tests and *η^2^* for ANOVAs.

## Results

### Experiment 1: ERPs Elicited by Pitch-Deviant Auditory Feedback

**FIGURE 2:**
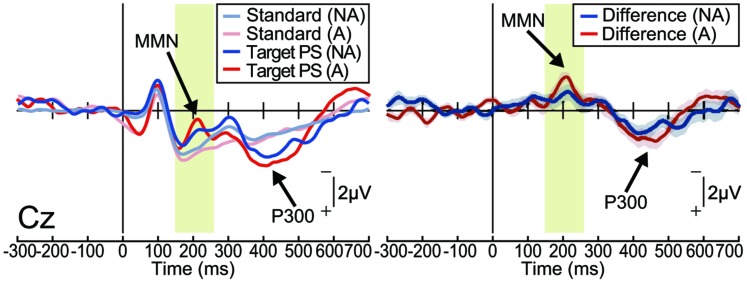
**Pitch-deviant auditory feedback elicited MMN and P300 (Experiment 1).** Left: the event-related potentials (ERPs) at Cz elicited by pitch-deviant stimuli presented passively (non-action condition; NA) or by a mouse-click performed by the participant (action condition; A; *n* = 16). Right: differential (target – standard) ERP waveforms. Shaded (blue or red) areas represent SEM. The MMN was observed around 200 ms from the stimulus onset, both in the non-action [*t*_(15)_ = 4.84, *P* < 0.001, effect size *r* = 0.78] and action conditions [*t*_(15)_ = 6.20, *p* < 0.001, effect size *r* = 0.85]. The P300 followed the MMN [non-action: *t*_(15)_ = 9.07, *p* < 0.001, effect size *r* = 0.92; action: *t*_(15)_ = 6.02, *p* < 0.001, effect size *r* = 0.84].

In the non-action condition, the target stimulus elicited a MMN in the frontocentral area [one-sample *t*-test; Fz: *t*_(15)_ = 3.88, *p* < 0.001, effect size *r* = 0.72; Cz: *t*_(15)_ = 4.84, *p* < 0.001, effect size *r* = 0.78; **Figure 2** and Supplementary Figure S1]. The target stimulus also elicited a P300, which is considered to reflect conscious detection of the target stimulus (Duncan-Johnson and Donchin, 1982; Picton, 1992), for latencies ranging from 250 to 450 ms [Cz: *t*_(15)_ = 9.07, *p* < 0.001, effect size *r* = 0.92]. These results are consistent with those of previous studies in which passively presented pitch-deviant stimuli elicited both MMN and P300 components (Joos et al., 2014). Similar results were obtained in the action condition [MMN at Cz: *t*_(15)_ = 6.20, *p* < 0.001, effect size *r* = 0.85; P300 at Cz: *t*_(15)_ = 6.02, *p* < 0.001, effect size *r* = 0.84; **Figure 2** and Supplementary Figure S1]. The latencies and amplitudes of the MMN and P300 at each electrode were equivalent between the action and non-action conditions (paired *t*-test; *p* > 0.1, effect size *r* < 0.35; Supplementary Figures S1B–D). These results demonstrate that the pitch-deviant stimulus elicited the MMN and P300 both when the stimulus was generated passively and when it was produced by participant self-movement.

### Experiment 2: ERPs Elicited by Delayed Auditory Feedback

**FIGURE 3:**
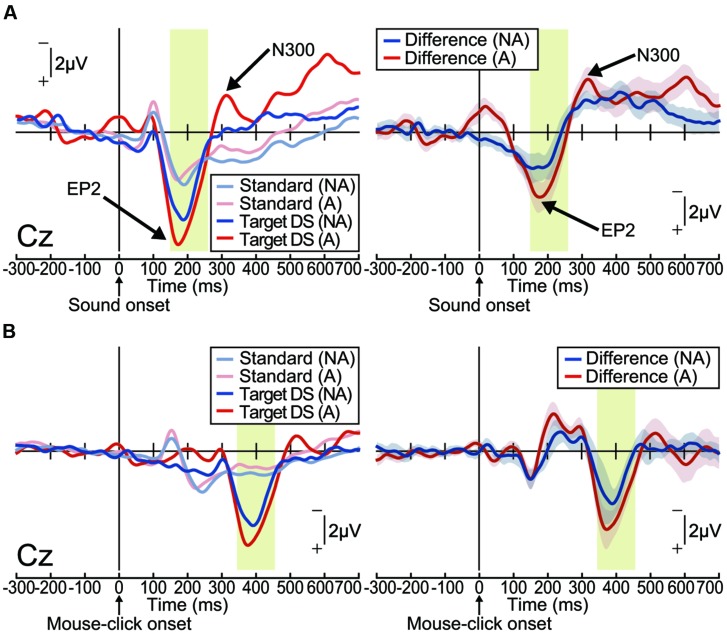
**Delayed auditory feedback elicited an enhanced-P2 (EP2) and N300 (Experiment 2).** Grand-averaged ERP waveforms computed by **(A)** auditory stimulus onset or **(B)** action (mouse-click performed by participant) onset. **(A)** Left: ERPs elicited by passive presentation of a delayed (150 ms) auditory stimulus (non-action condition; NA) or elicited by the mouse-click action performed by the participant (action condition; A; *n* = 16). Right: differential (target – standard) ERP waveforms. Shaded (blue or red) areas represent SEM. The EP2 (positive peak around 200 ms) was apparent in both conditions [non-action: *t*_(15)_ = 4.89, *p* < 0.001, effect size *r* = 0.79; action: *t*_(15)_ = 5.99, *p* < 0.001, effect size *r* = 0.84]. The N300 (negative peak around 300 ms) followed the EP2 [non-action: *t*_(15)_ = 4.30, *p* < 0.001, effect size *r* = 0.74; action: *t*_(15)_ = 4.71, *p* < 0.001, effect size *r* = 0.77]. **(B)** Left: ERPs elicited by passive presentation (NA) of a delayed (150 ms) auditory stimulus or by a mouse-click performed by the participant (A; *n* = 16). Right: differential (target – standard) ERP waveforms. Shaded (blue or red) areas represent SEM.

The ERP waveforms elicited by the delayed auditory stimulus were strikingly different from those observed during the pitch-deviant oddball task in Experiment 1 (**Figure 3A**). In both the action and non-action conditions, delayed stimuli elicited a positive deflection within a latency of around 200 ms, which was most prominent at the centroparietal area [one-sample *t*-test; Non-action condition at Cz: *t*_(15)_ = 4.89, *p* < 0.001, effect size *r* = 0.79; Action condition at Cz: *t*_(15)_ = 5.99, *p* < 0.001, effect size *r* = 0.84; Supplementary Figure S2A]. We refer to this differential (deviant – standard) ERP component as ‘enhanced-P2,’ as it seems that P2, which is an ERP component that is robustly elicited by an auditory stimulus, was amplified (Gregg and Snyder, 2012). Notably, the enhanced-P2 had a similar latency to the MMN in Experiment 1, but with an opposite (positive) polarity. The delay-deviant stimulus also elicited a negative deflection at approximately 300 ms after the stimulus onset [non-action condition at Cz: *t*_(15)_ = 4.30, *p* < 0.001, effect size *r* = 0.74; action condition at Cz: *t*_(15)_ = 4.71, *p* < 0.001, effect size *r* = 0.77; **Figure 3A** and Supplementary Figure S2A). We shall refer to this component as N300.

To assess the conditional differences (action vs. non-action), we analyzed the latency and amplitude of the enhanced-P2 and N300 via two-tailed paired *t*-tests. The latency and amplitude of the enhanced-P2 and N300 at each electrode were equivalent between the action and non-action conditions (paired *t*-test; *p* > 0.2, effect size *r* < 0.31; Supplementary Figures S2B–E).

We then analyzed action-locked (mouse-click onset) waveforms (**Figure 3B** and Supplementary Figure S2F). Focusing on the ERP waveforms before the action onset, we did not find any motor-related negative slope resembling a readiness potential (Libet et al., 1983) or a contingent negative variation (CNV; Walter et al., 1964; Kornhuber and Deecke, 1965). Instead, we found negative peaks at around 200 ms after the mouse-click onset (one-sample *t*-test; *p* < 0.05, effect size *r* > 0.44; **Figure 3B** and Supplementary Figures S2G,H). This seems like the omission MMN, which is elicited when an expected stimulus is omitted in an oddball experimental paradigm (Raij et al., 1997; May and Tiitinen, 2010; Todorovic et al., 2011; SanMiguel et al., 2013). However, we do not further discuss this issue here since the current experiment was not designed to examine the omission MMN, as the stimulus was only delayed, not omitted. In any case, the ERP components of interest (enhanced-P2 and N300) were elicited after this negative peak (**Figure 3B**). We thus consider that these ERP components have been triggered by the associated delayed auditory feedback, and not by the self-generated action *per se*.

### Experiment 3: Functional Dissociation of ERPs Elicited by Pitch-Deviant and Delayed Auditory Feedback

#### Behavioral Data

The average number of target stimuli counted by participants was 180.9 ± 9.4 in the attend-to-pitch session and 154.3 ± 18.2 in the attend-to-delay session, where the target stimulus (deviant stimulus of interest) was presented 180 times (90 times each for PS, DS, and PDS). We found a significant difference between sessions [*t*_(15)_ = 8.50, *p* < 0.001, effect size *r* = 0.91], indicating that delay-deviant stimulus detection was more difficult than pitch-deviant stimulus detection in this experiment. However, the number of deviant stimuli detected was adequately large (>85%), so we considered the participants to have effectively attended to the stimuli in both experimental sessions.

#### ERP Results

**FIGURE 4:**
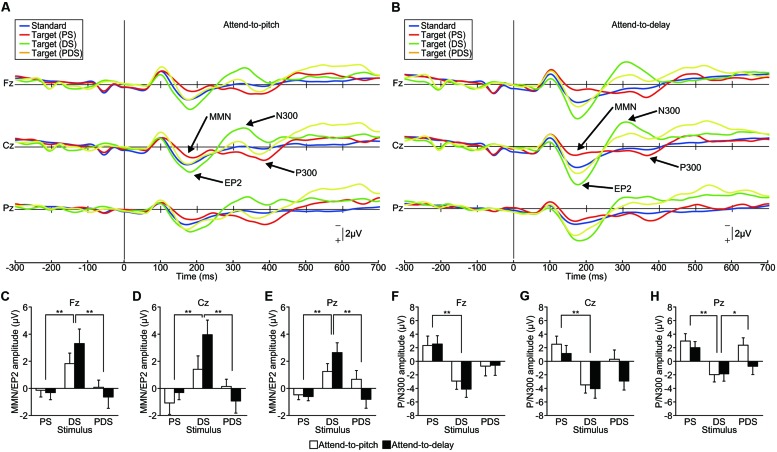
**Functional dissociation between the EP2/N300 and MMN/P300 (Experiment 3). (A,B)** Grand-averaged ERP waveforms elicited by pitch-deviant (PS), delayed (DS), and pitch-deviant delayed (PDS) feedback (action conditions only, *n* = 16). The participant was instructed to attend to either **(A)** pitch-deviant (PS and PDS) or **(B)** delayed (DS and PDS) auditory feedback. **(C–H)** The amplitude of the MMN/EP2 and P/N300 at each electrode. **(C–E)** Two-way repeated-measures ANOVAs examining the amplitude of the MMN/EP2 revealed a significant main effect of the type of feedback [Fz: *F*_(1,90)_ = 9.14, *p* < 0.01, effect size η^2^ = 0.17; Cz: *F*_(1,90)_ = 9.31, *p* < 0.001, effect size η^2^ = 0.17; Pz: *F*_(1,90)_ = 10.08, *p* < 0.001, effect size η^2^ = 0.18] but no main effect of selective attention (*p* > 0.3). A *post hoc* analysis (Tukey’s HSD test) revealed significant differences between PS and DS and between DS and PDS at all electrodes (*p* < 0.01). **(F–H)** Two-way repeated-measures ANOVAs examining the amplitude of the P/N300 revealed significant main effects of the type of feedback [Fz: *F*_(1,90)_ = 9.25, *p* < 0.001 effect size η^2^ = 0.17; Cz: *F*_(1,90)_ = 9.01, *p* < 0.001 effect size η^2^ = 0.17; Pz: *F*_(1,90)_ = 8.02, *p* < 0.001 effect size η^2^ = 0.15]. A *post hoc* analysis (Tukey’s HSD test) revealed significant differences between PS and DS at all electrodes (*p* < 0.01) and between DS and PDS at Pz (*p* < 0.05). ^∗^*p* < 0.05, ^∗∗^*p* < 0.01. Error bars represent SEM.

Grand-averaged ERP waveforms are shown in **Figures 4A,B** (see also Supplementary Figure S3). A PS elicited an MMN (dominant at Fz) and a P300 (dominant at Pz) as in Experiment 1, and a DS elicited an enhanced-P2 and an N300, both dominant at Cz. In contrast, a PDS failed to elicit either MMN/enhanced-P2 or P/N300. We compared the latency and amplitude of these ERP components among the stimuli (PS, DS, vs. PDS) and sessions (attend-to-pitch vs. attend-to-delay) using repeated-measure two-way ANOVAs. For the ERP located around 200 ms (MMN/enhanced-P2), we found no effect of session on either the latency or amplitude at any electrode (*p* > 0.1), with one exception: we observed an effect of session on the MMN/enhanced-P2 latency at Pz [*F*_(1,95)_ = 5.65, *p* < 0.05, effect size η^2^ = 0.06). This indicates that selective attention did not affect the MMN and enhanced-P2. As the latencies of the MMN/enhanced-P2 at Pz were significantly different between sessions, this result demonstrates that MMN/enhanced-P2 are functionally different components. In contrast, we found that the stimuli greatly affected the amplitude of the enhanced-P2 at all electrodes (Fz, Cz, and Pz, *p* < 0.001, effect size η^2^ > 0.17; **Figures 4C–E**). We found no significant interactions between stimuli and sessions (*p* > 0.5, effect size η^2^ < 0.06). A subsequent Tukey’s HSD test revealed significant differences in amplitude between PS and DS and between DS and PDS at all electrodes (*p* < 0.01). This result indicates that the pitch-deviant stimulus elicited a MMN while the delay-deviant stimulus elicited an enhanced-P2, irrespective of selective attention.

Similarly, the ERP amplitude around 300 ms (P/N300) showed effects of stimuli at all electrodes (*p* < 0.001, effect size η^2^ > 0.14; **Figures 4F–H**). Subsequent analyses (Tukey’s HSD) revealed significant differences between PS and DS at all electrodes (*p* < 0.01) and between DS and PDS at Pz (*p* < 0.05), indicating that the pitch-deviant stimulus elicited a P300 while the delayed stimulus elicited a N300. We did not find an effect of session (attention) on the amplitude of the P/N300 (*p* > 0.1, effect size η^2^ < 0.03).

Our results demonstrate that ERP components elicited by pitch-deviant and delayed auditory stimuli are essentially different from each other. PDS failed to elicit any deviant-related ERPs, probably owing to a superposition effect between the ERPs elicited by pitch-deviant feedback and ERPs elicited by delayed feedback. This supports the hypothesis that pitch-deviant stimuli and delayed auditory feedback are processed by different neural mechanisms. This experimental design excludes any contamination from factors of non-interest, as these results were obtained from single experimental runs with the same (within) subjects.

### Experiment 4: Sensitivity to Delay Length

#### Behavioral Data

**FIGURE 5:**
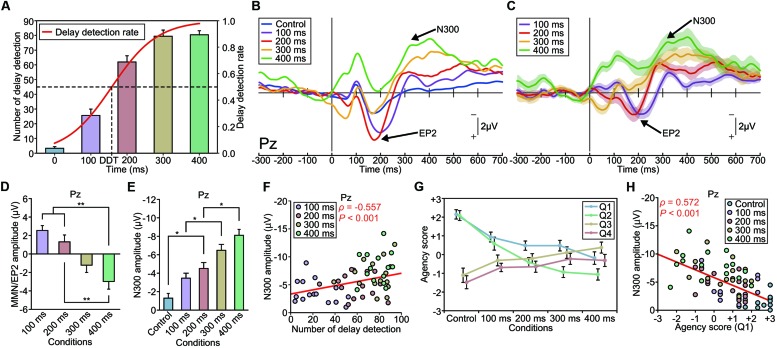
**Modulation of the EP2 and N300 by delay length (Experiment 4). (A)** The number of auditory feedback delays that the participant detected increased as a function of the delay length. Delay detection probabilities were fitted to a logistic function. The delay detection threshold (DDT), where the probability is 50%, was estimated to be 158 ms. **(B)** Grand-averaged ERP waveforms at Pz (*n* = 16). Deviant auditory feedback was delayed by either 0 ms (control), 100, 200, 300, or 400 ms, with each delay size presented in a separate session. **(C)** Differential (deviant – standard) ERP waveforms. Shaded areas represent SEM. **(D)** The amplitude of the EP2 (MMN) was significantly modulated by the delay length. A one-way ANOVA revealed a main effect of delay [Pz: *F*_(3,60)_ = 10.70, *p* < 0.001, effect size η^2^ = 0.35]. A *post hoc* analysis (Tukey’s HSD test) revealed a significant difference between the 100/200-ms-delay and 400-ms-delay conditions. Notably, the P2 was enhanced in the shorter-delay (≤200 ms) conditions while the MMN was present in the longer-delay (≥300 ms) conditions. **(E)** The amplitude of the N300 was significantly modulated by the delay length [Pz: *F*_(4,75)_ = 18.30, *p* < 0.001, effect size η^2^ = 0.49]. A *post hoc* analysis (Tukey’s HSD test) indicated that the absolute amplitude of the N300 was significantly larger in the 300 and 400-ms-delay conditions compared with the other conditions. **(F)** The N300 amplitude was significantly correlated with the rate of subjective detection of the auditory feedback delay. A correlation analysis showed a significant correlation between the amplitude of the N300 at Pz and the counted number of delayed stimuli (ρ = -0.557, *p* < 0.001, Spearman’s rank correlation coefficient). **(G)** We assessed the sense of agency via a questionnaire: Q1 and Q2 concerned the sense of agency, while Q3 and Q4 served as dummy questions (see Materials and Methods). The sense of agency was attenuated as the delay length increased. A one-way ANOVA revealed a significant effect of the delay [Q1: *F*_(4,75)_ = 9.22, *p* < 0.001, effect size η^2^ = 0.33; Q2: *F*_(4,75)_ = 18.91, *p* < 0.001, effect size η^2^ = 0.50]. A *post hoc* analysis exposed significant differences between the shorter-delay conditions and the longer-delay conditions. **(H)** A correlation analysis revealed a significant correlation between the amplitude of the N300 at Pz and the agency score (Q1: ρ = 0.572, *p* < 0.001, Spearman’s rank correlation coefficient). A similar result was obtained for Q2 (ρ = 0.545, *p* < 0.001). ^∗^*p* < 0.05, ^∗∗^*p* < 0.01. Error bars represent SEM.

The average number of deviant stimuli counted by the participants monotonically increased from the control (non-delayed) to the 300-ms-delayed condition [one-way ANOVA, *F*_(4,75)_ = 87.65, *p* < 0.001, effect size η^2^ = 0.82; **Figure 5A**). Subsequent analyses (Tukey’s HSD test) showed that there were significant differences in the counted number between every pair of conditions (*p* < 0.01), except between the 300 and 400-ms-delayed conditions (*p* = 0.999). This result indicates that the participants detected more delayed stimuli as the length of the delay increased. We calculated the DDT, which was estimated as 158 ms in this experiment (**Figure 5A**).

#### ERP Results

Grand-averaged ERP waveforms are shown in **Figures 5B,C** and Supplementary Figures S4A–D,K,L. While the enhanced-P2 was observed in the 100 and 200-ms-delayed conditions, as in the previous experiments; it was not present in the 300 and 400-ms-delayed conditions. A one-way ANOVA revealed a significant effect of condition on the amplitude of the enhanced-P2 at Pz [*F*_(3,60)_ = 10.70, *p* < 0.001, effect size η^2^ = 0.35] (**Figure 5D** and Supplementary Figures S4E–G). A subsequent Tukey’s HSD test revealed significant differences between the 100 and 200-ms-delayed condition and the 400-ms-delayed conditions and between the 200-ms-delayed condition and the 400-ms-delayed condition (*p* < 0.01; **Figure 5D**). Importantly, the delay length critically affected the polarity of the differential responses. Specifically, enhancement of the P2 was apparent in the 100–200-ms conditions, but absent (i.e., MMN was observed instead; **Figures 5B,C**) in the 300–400-ms conditions.

Interestingly, the N300 showed a considerable modulation between conditions, but in a substantially different manner from that of the enhanced-P2/MMN (**Figure 5E** and Supplementary Figures S4H–J). A repeated-measure one-way ANOVA revealed that the amplitude of the N300 was significantly different among conditions at Pz [*F*_(4,75)_ = 18.30, *p* < 0.001, η^2^ = 0.49] and Cz [*F*_(4,75)_ = 5.74, *p* < 0.001, η^2^ = 0.24]. A subsequent Tukey’s HSD test revealed that the N300 at Pz was significantly greater in the 200-ms-delayed condition compared with the control condition, greater in the 300-ms-delayed condition compared with the 100-ms-delayed condition, and greater in the 400-ms-delayed condition compared with the 200-ms-delayed condition (*p* < 0.05; **Figure 5E**). Similarly, the N300 at Cz was significantly greater in the 300 and 400-ms-delayed conditions compared with the control condition (*p* < 0.01). The latency of the N300 was not significantly different among conditions at each electrode (*p* > 0.1; effect size η^2^ < 0.08).

Regression analyses revealed that the N300 amplitude increased linearly as a function of the delay length at Pz (*R*^2^ = 0.992) and Cz (*R*^2^ = 0.940). Similar results were obtained for the enhanced-P2 at Pz (*R*^2^ = 0.985) and Cz (*R*^2^ = 0.830).

### Correlation Between the Amplitude of the N300 Component and the Sense of Agency

Our result that the N300 monotonically increased as a function of the delay length (**Figure 5E**) is consistent with our behavioral result regarding the counted delayed stimuli. Indeed, we found a significant correlation between the amplitude of the N300 at Pz and the counted number of delayed stimuli (ρ = -0.557, *n* = 64, *p* < 0.001, Spearman’s correlation coefficient; **Figure 5F**), indicating that the N300 amplitude became larger as the participant detected the delayed stimuli more frequently. We also assessed the sense of agency, which is the feeling of authorship of the action, for each condition via a questionnaire (see Materials and Methods). The sense of agency was attenuated as the length of the delay increased [*F*_(4,75)_ = 9.22, *p* < 0.01, effect size η^2^ = 0.33, for Q1; *F*_(4,75)_ = 18.91, *p* < 0.01, effect size η^2^ = 0.50, for Q2; **Figure 5G**]. Subsequent analyses (Tukey’s HSD tests) indicated that the agency score was significantly weaker in all delay conditions compared with the control condition, as assessed by Q1, and that the agency score was weaker in the 200, 300, and 400-ms-delayed conditions compared with the control and 100-ms-delayed conditions, as measured by Q2 (*p* < 0.05). The N300 amplitude at Pz was significantly correlated with the magnitude of the sense of agency (ρ = 0.572, *n* = 80, *p* < 0.001, for Q1; **Figure 5H**; ρ = 0.545, *n* = 80, *p* < 0.001, for Q2, Spearman’s correlation coefficient); that is, the N300 amplitude was greater when the subjective sense of agency was weakened. We found no significant correlation between the mean amplitude of enhanced-P2 and the agency score (ρ < 0.17, *p* > 0.2), between the peak latency of enhanced-P2 and the agency score (ρ < 0.13, *p* > 0.3), or between the peak latency of N300 and the agency score (ρ < 0.09, *p* > 0.4) at Pz. These results demonstrate that the N300 amplitude strongly reflects the subjective detection of delayed auditory feedback, and hence an attenuated sense of agency.

## Discussion

We have demonstrated that the perception of delayed auditory feedback of self-generated movement elicits early ERP components, namely the enhanced-P2 and N300 (Experiment 2), which are apparently different from the MMN and P300 observed in the pitch-deviant oddball paradigm (Experiment 1). The findings from our mixed design experiment clearly show that the enhanced-P2/N300 and MMN/P300 differ functionally (Experiment 3). Further, these ERP components were significantly modulated by the length of an auditory feedback delay (Experiment 4). The amplitude of the N300 increases as a function of the delay length, and is positively correlated with subjective delay detection. In contrast, the enhanced-P2 is observed only in shorter (≤200 ms) delay conditions, while the MMN is only observed in longer (≥300 ms) delay conditions. Our results suggest that different neural mechanisms are employed for the processing of temporally deviant and pitch-deviant auditory feedbacks. Accordingly, we consider that the temporal window for motor–auditory temporal integration is about 200 ms, manifested in these early ERP components.

We found that the N300 was robust in response to delayed stimuli, and was most prominent at the centroparietal area. Additionally, the amplitude of the N300 was strongly correlated with the conscious detection of the delay. The N300 has been observed in a variety of cognitive tasks where an individual is presented with a stimulus that violates their prediction, such as visual object identification (Demiral et al., 2012; Mudrik et al., 2014) and semantic word categorization (Renoult et al., 2012). Renoult showed that stimulus repetition shortened the latency of the N400 from around 380 to 340 ms, and postulated that the N300 is functionally identical to the N400. The N400 is a well-known ERP component that is mainly related to language processing (Kutas and Hilyard, 1980). However, it has recently been linked to a wider range of meaning processing, including visual, auditory, and action recognition (Reinke et al., 2003; Kutas and Federmeier, 2011; Stekelenburg et al., 2011; Sun et al., 2012). An intere–sting characteristic of the N300–N400 is that its amplitude is sensitive to the magnitude of a deviation, while its latency is kept relatively constant. Our finding that delay length is a critical factor in the modulation of N300 amplitude, but not latency, is in accordance with previous studies.

Interestingly, the correlation between N300 amplitude and the agency score was most prominent at the parietal electrode (Pz). This suggests parietal involvement in the sense of agency. Indeed, previous studies have repeatedly shown that the parietal lobe is critically related to the sense of agency. For instance, Farrer et al. (2003) reported graded activation in the right angular gyrus when spatial distortion of a self-generated movement was gradually enlarged. Additionally, our previous study reported parietal involvement in the sense of self-body ownership (Shimada et al., 2005). A recent study found that parietal and premotor cortices are involved in bringing motor intentions and motor responses into awareness (Desmurget et al., 2009). Although precise source localization of the N300 is required, and is thus a subject for future study, it is likely that the parietal lobe is critically involved in multisensory integration processes regarding the sense of agency.

Contrary to our initial expectations, we failed to find the MMN in the delay (≤200 ms) conditions, and instead found the enhanced-P2. Previous studies have shown that the P2 can be enhanced by training and expertise (Shahin et al., 2003; Baumann et al., 2008) but not by selective attention (Amoruso et al., 2013). Several recent studies have shown that enhancement of the P2 is also involved in the processing of deviant stimuli. One study reported that P2-enhancement was observed when auditory stimuli that were slightly different were presented successively (separated by white noise), especially when the participant was not aware of the change (‘change deafness’). In contrast, change detection is well reflected by the P1 and P300 (Gregg and Snyder, 2012). Another study showed that a slight shift in the pitch of auditory feedback about self-vocalizations elicited strong enhanced-P2 compared with those elicited by passive listening to the playback of the vocalization (Chen et al., 2013). Finally, a recent study showed that the N1-P2 component is related to the processing of temporal deviance in auditory stimuli (Kononowicz and van Rijn, 2014). In the above-mentioned study, participants compared the duration of a sound stimulus lasting approximately 1600–3000 ms with that of a standard stimulus that lasted for 2200 ms. The authors found that the amplitude of the N1-P2 elicited by the offset of the stimulus increased as the temporal difference between the stimuli was lengthened. Considering the findings of previous reports, along with our result that DDT was 158 ms (Experiment 4), our work suggests that the enhanced-P2 is related to the processing of deviant auditory stimuli that are nearly equal to the threshold of conscious detection or that have been the focus of substantial attention.

Interestingly, we only observed the enhanced-P2 in response to 100 and 200-ms-delayed auditory feedback, and not in delayed conditions ≥300 ms. Alternatively, we observed the MMN in the longer delay conditions. Considering the characteristics of the enhanced-P2 and N300 described above, a shorter feedback delay elicits implicit effort to process subthreshold stimuli, while a longer delay leads to the conscious detection of a regularity violation. Our results indicate that delay length is a critical factor in the differential elicitation of early auditory-processing ERP components that reflect the implicit integration of multisensory inputs (enhanced-P2) or the conscious detection of a deviant stimulus (N300). Additionally, the border of this delay length appears to lie somewhere between 200 and 300 ms.

Indeed, a delay of 200–300 ms is critical duration for self-body or self-generated movement recognition, namely, the sense of ownership and the sense of agency (Gallagher, 2000, 2005). For example, Blakemore et al. showed that tickliness in response to a self-generated stimulus was elevated as the delay between the tactile sensation and the self-action increased up to 300 ms (Blakemore et al., 1999). Additionally, temporal order judgment of tactile stimulations applied to the right and left hands can be confused (reversed) with temporal intervals of less than 300 ms, especially when the arms are crossed (Yamamoto and Kitazawa, 2001; Miyazaki et al., 2006). Shimada et al. (2009, 2014) demonstrated that the magnitude of the rubber hand illusion (RHI), which is an illusion regarding self-body attribution, was attenuated as the temporal discrepancy between visual and tactile stimulation was increased. The authors showed that the RHI decreased when a visual feedback delay was greater than 200–300 ms. Finally, several studies addressing the sense of ownership and agency during delayed sensory feedback have consistently reported a threshold around 200–300 ms (Shimada et al., 2005, 2010; Toida et al., 2014). Cumulatively, these findings suggest that there is a temporal window for integrating self-body or self-movement information, with a time constant of 200–300 ms, in the human brain. Our study indicates that the early ERP components, specifically enhanced-P2 and N300, are useful measures for further investigation of the neural mechanisms that underlie multisensory integration with respect to self-body and self-movement.

## Conflict of Interest Statement

The authors declare that the research was conducted in the absence of any commercial or financial relationships that could be construed as a potential conflict of interest.
