# Supplementary material for: Neural Basis of the Time Window for Subjective Motor-Auditory Integration
Source: Front Hum Neurosci. 2016 Jan 7;9:688. doi: 10.3389/fnhum.2015.00688 (PMC4704610; doi:10.3389/fnhum.2015.00688)
Supplement: Supplementary file 1 [file Image_1.PDF]

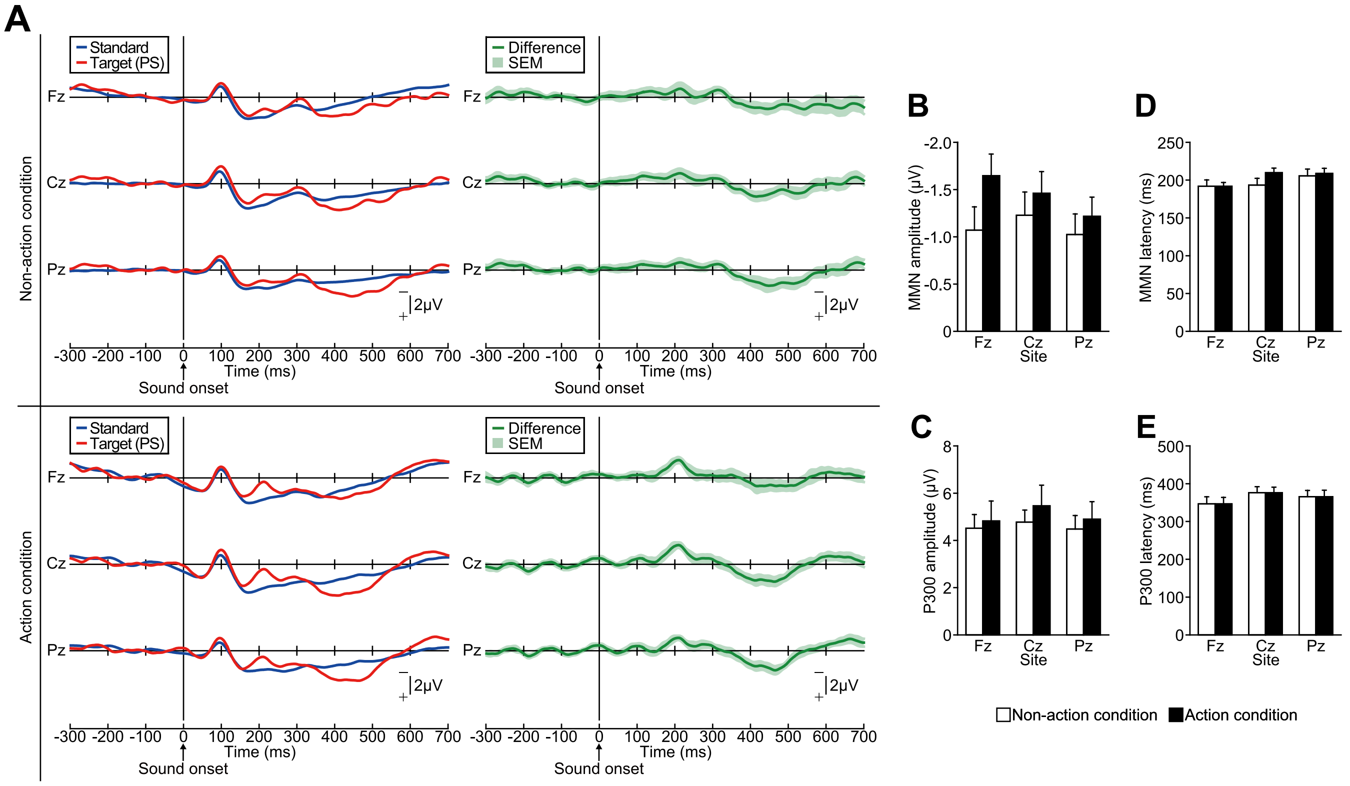

SUPPLEMENTARY FIGURE S1 | Pitch-deviant auditory feedback elicits MMN and P300 components (Experiment 1). (A) Left: the event-related potentials (ERPs) elicited by a passively presented pitch deviant stimulus (non-action condition; top) or by a mouse-click performed by the participant (action condition; bottom) (n = 16). Right: differential (deviant − standard) ERP waveforms. Shaded (green) areas represent SEM. The MMN was observed frontocentrally (Fpz-Cz) around 200 ms from the stimulus onset in both the non-action and action conditions. The P300 followed the MMN. **(B-E)** The amplitude and latency of the MMN and P300 at each electrode. Error bars represent SEM.
