# Supplementary material for: Neural Basis of the Time Window for Subjective Motor-Auditory Integration
Source: Front Hum Neurosci. 2016 Jan 7;9:688. doi: 10.3389/fnhum.2015.00688 (PMC4704610; doi:10.3389/fnhum.2015.00688)
Supplement: Supplementary file 2 [file Image_2.PDF]

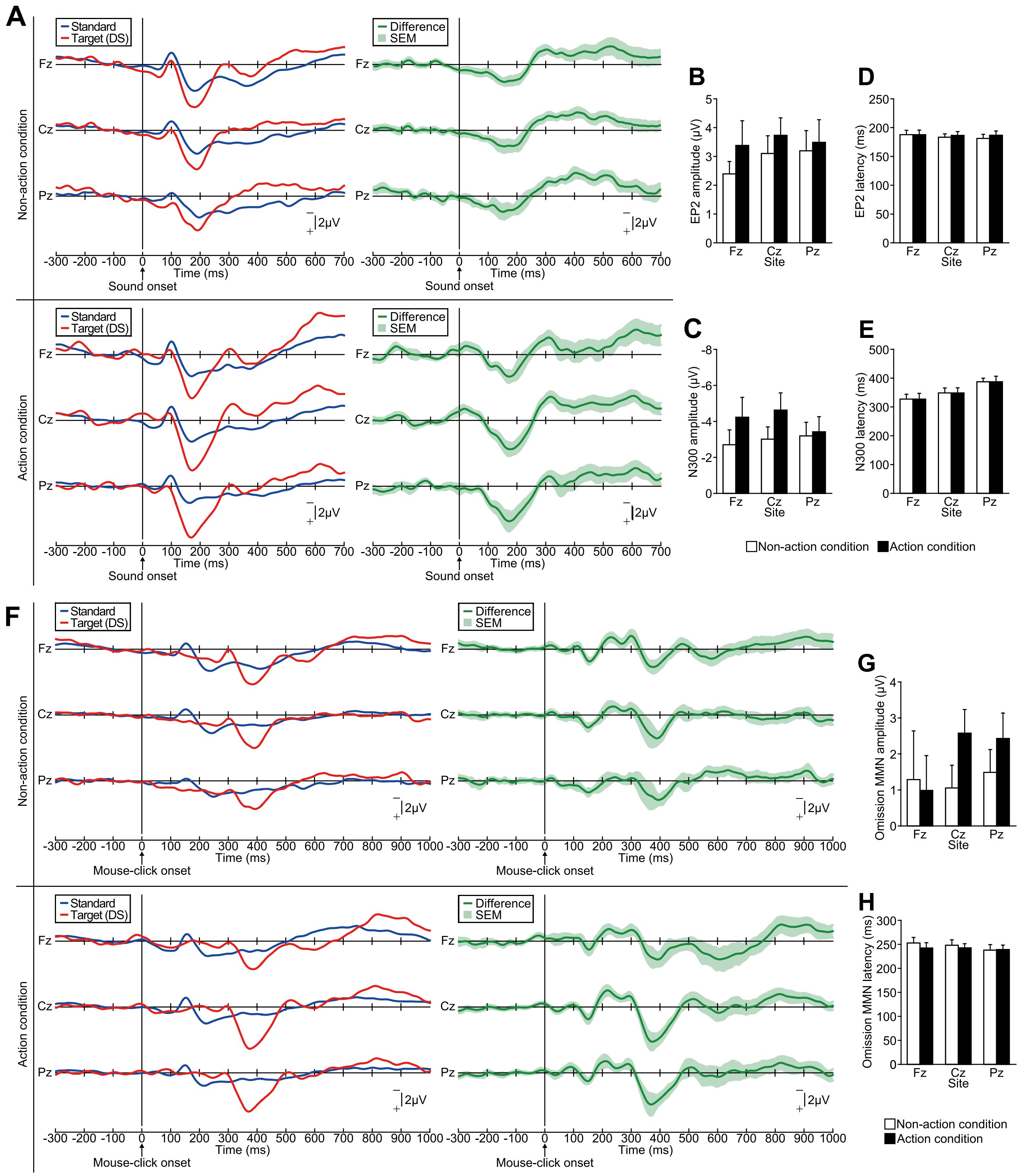

SUPPLEMENTARY FIGURE S2 | Delayed auditory feedback elicits an enhanced-P2 (EP2) and an N300 (Experiment 2). Grand-averaged ERP waveforms computed by **(A)** auditory stimulus onset or **(F)** action (a mouse-click performed by the participant) onset. **(A)** Left: ERPs elicited by the passively presented delayed (150 ms) auditory stimulus (non-action condition; top) or by a mouse-click performed by the participant (action condition; bottom) (n = 16). Right: differential (deviant − standard) ERP waveforms. Shaded (green) areas represent SEM. The EP2 (positive peak around 200 ms) was apparent in both conditions. The N300 (negative peak around 300 ms) followed the EP2. **(B-E)** The amplitude and latency of the EP2 and N300 at each electrode. Error bars represent SEM. **(F)** Left: ERPs elicited by the passively presented delayed (150 ms) auditory stimulus (non-action condition; top) or by a mouse-click performed by the participant (action condition; bottom) (n = 16). Right: differential (deviant − standard) ERP waveforms. Shaded (green) areas represent SEM. The omission MMN (positive peak at around 100 ms) was apparent in both conditions ($p < 0.01$). The N300 (negative peak at around 300 ms) followed the EP2 ($p < 0.001$). **(G,H)** The amplitude and latency of the omission MMN at each electrode. Error bars represent SEM.
