# Supplementary material for: Neural Basis of the Time Window for Subjective Motor-Auditory Integration
Source: Front Hum Neurosci. 2016 Jan 7;9:688. doi: 10.3389/fnhum.2015.00688 (PMC4704610; doi:10.3389/fnhum.2015.00688)
Supplement: Supplementary file 3 [file Image_3.PDF]

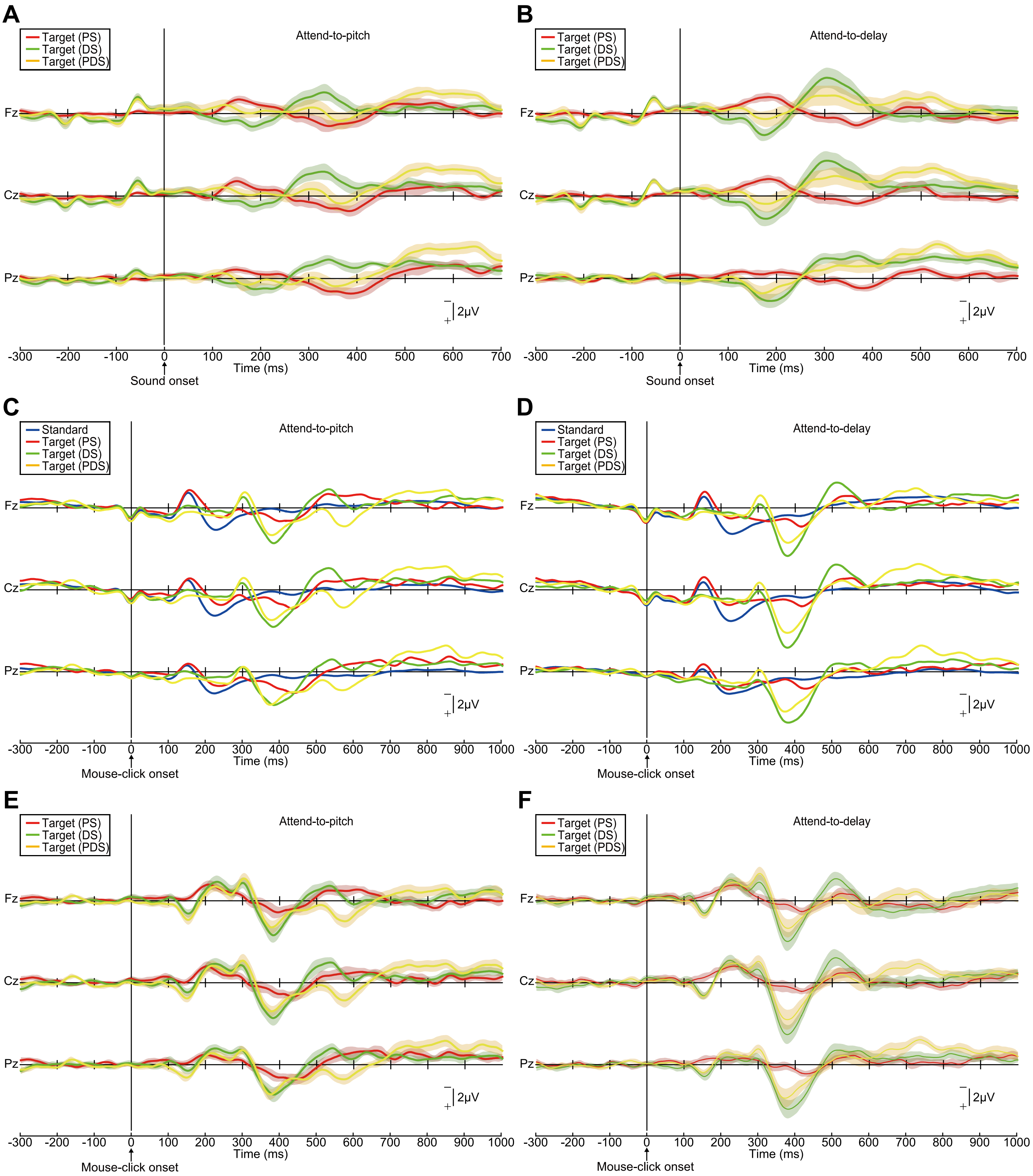

SUPPLEMENTARY FIGURE S3 | Functional dissociation of the EP2/N300 and MMN/P300 (Experiment 3). (A,B) Differential (deviant – standard) ERP waveforms computed by auditory stimulus onset for pitch-deviant (PS), delayed (DS), and pitch-deviant delayed (PDS) feedback (action conditions only, *n* = 16). **(C,D)** Grand-averaged ERP waveforms computed by action (participants' mouse-click) onset. **(E,F)** Differential ERP waveforms computed by action onset. Shaded (red, green and yellow) areas represent SEM. The participant was instructed to attend to either the pitch-deviant **(A,C,E)** or delayed feedback **(B,D,F)**.
