# Supplementary material for: Neural Basis of the Time Window for Subjective Motor-Auditory Integration
Source: Front Hum Neurosci. 2016 Jan 7;9:688. doi: 10.3389/fnhum.2015.00688 (PMC4704610; doi:10.3389/fnhum.2015.00688)
Supplement: Supplementary file 4 [file Image_4.PDF]

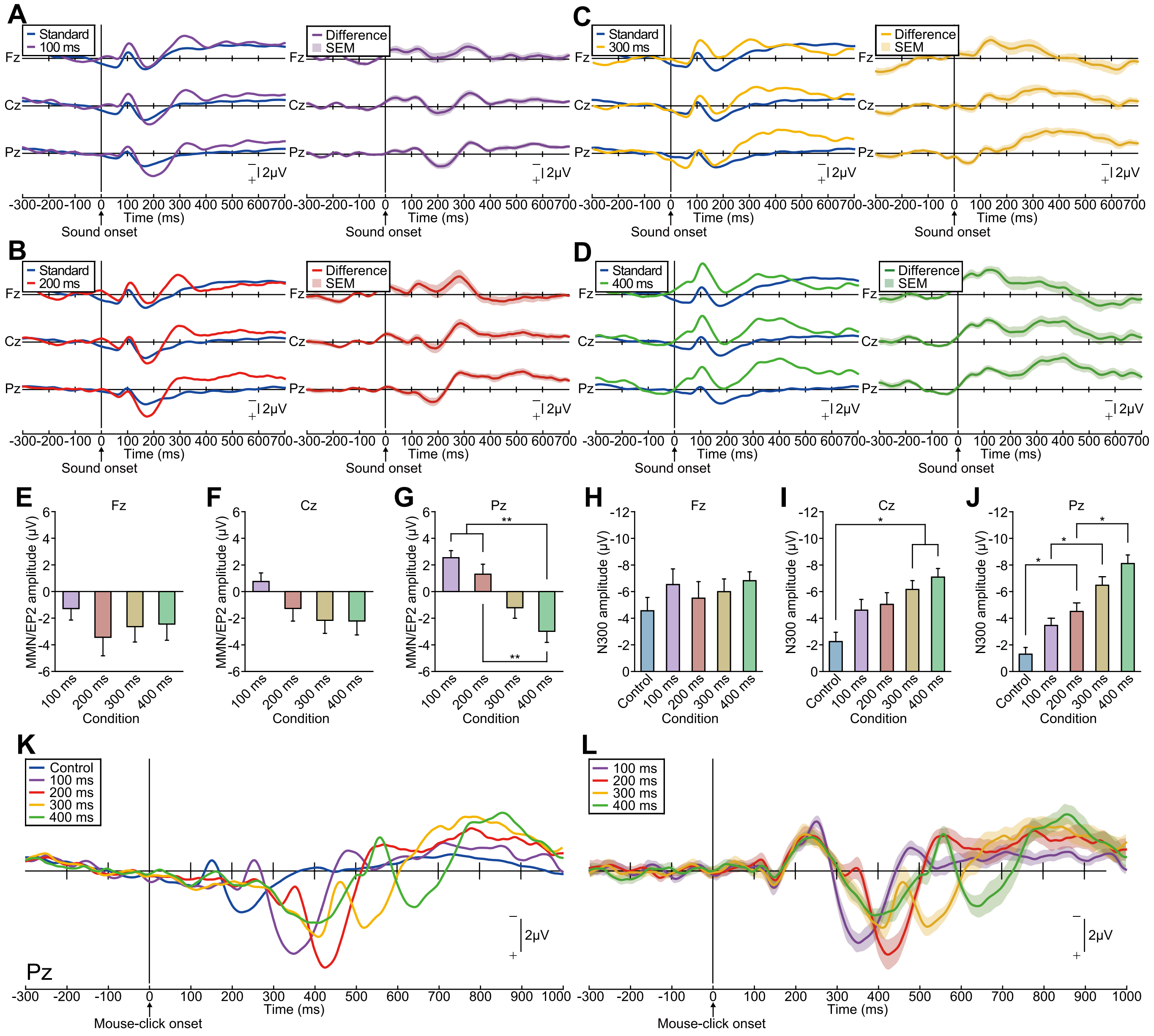

SUPPLEMENTARY FIGURE S4 | Modulation of the EP2 and N300 by the delay length (Experiment 4). (A–D) Left: grand-averaged ERP waveforms (auditory stimulus onset) (*n* = 16). Right: differential (deviant − standard) ERP waveforms. Shaded areas represent SEM. The deviant auditory feedback was delayed by either **(A)** 100 ms, **(B)** 200 ms, **(C)** 300 ms, or **(D)** 400 ms, with each delay length presented in separate sessions. **(E-G)** The amplitude of the EP2 (MMN) was significantly modulated by the delay length. A one-way ANOVA exposed a main effect of delay. A post hoc analysis (Tukey' s HSD test) revealed a significant difference between the 100/200-ms-delay and 300/400-ms-delay conditions. Notably, the enhancement of P2 was present in the shorter (≤ 200 ms) delay conditions while the MMN was observed in the longer (≥ 300 ms) delay conditions. **(H-J)** The amplitude of the N300 was significantly modulated by delay length in the parieto-central regions. A post hoc analysis (Tukey' s HSD test) demonstrated that the absolute amplitude of the N300 was significantly larger in the 300-ms- and 400-ms-delay conditions compared with the other conditions. **(K,L)** Left; grand-averaged ERP waveforms (mouse-click onset) (*n* = 16). Right; differential (deviant − standard) ERP waveforms. Shaded areas represent SEM. *$p < 0.05$, **$p < 0.01$. Error bars represent SEM.
